# Evaluation and pilot implementation of essential interventions for the management of hypertension and prevention of cardiovascular diseases in primary health care in the Republic of Tajikistan

**DOI:** 10.1186/s12913-021-06490-5

**Published:** 2021-05-18

**Authors:** Dylan Collins, Laura Inglin, Tiina Laatikainen, Mekhri Shoismatuloeva, Dilorom Sultonova, Bunafsha Jonova, Katoyon Faromuzova, Marifat Abdullaeva, Maisara Otambekova, Jill L. Farrington

**Affiliations:** 1 University of British Columbia, Vancouver, Canada; 2 Institute of Public Health and Clinical Nutrition, University of Eastern Finland, Kuopio, Finland; 3 Finnish Institute for Health and Welfare, Helsinki, Finland; 4 Joint municipal authority for North Karelia Health and Social Services, Joensuu, Finland; 5 World Health Organization Regional Office for Europe, Copenhagen, Denmark; 6 Service of State Supervision for Medical Activities and Social Protection of the Population of the Republic of Tajikistan, Dushanbe, Tajikistan; 7 Republican Clinical and Training Centre of Family Medicine, Dushanbe, Tajikistan; 8 Department of Epidemiology and Health Economics of the Faculty of Medicine, Tajik State University, Dushanbe, Tajikistan; 9 Public Health, Faculty of Medicine, Social Hygiene and Health Organization, Tajik State University, Dushanbe, Tajikistan

## Abstract

**Background:**

The aim of this study was to determine the feasibility of implementing and evaluating essential interventions for the management of hypertension and prevention of cardiovascular disease in primary healthcare in Tajikistan.

**Methods:**

The study protocol was published *a priori.* A pragmatic, sequential, mixed methods explanatory design was piloted. The quantitative strand is reported here. All primary health care facilities that met inclusion criteria in Shahrinav district were included and computer randomized to either usual care or intervention. The intervention consisted of: adaptation of WHO PEN/HEARTS clinical algorithms for hypertension and diabetes, a two-day training of doctors and nurses, supportive supervision visits, clinical decision support tools, and quality improvement support. Data were collected from paper-based clinical records at baseline and 12 months follow-up. The primary outcome was blood pressure control among patients with hypertension, in addition to several secondary process indicators along the care pathway. Age and sex adjusted logistic regression models were used for intervention and control clinics to determine changes between baseline and follow-up and to assess interactions between allocation group and time. For continuous variables, multivariate linear regression models were used.

**Results:**

19 primary health care centres were included of which ten were randomized to intervention and nine to control. 120 clinicians received training. The records of all registered hypertensive patients were reviewed at baseline and follow-up for a total of 1,085 patient records. Blood pressure control significantly improved in the intervention clinics (OR 3.556, 95 % CI 2.219, 5.696) but not the control clinics (OR 0.644, 95 % CI 0.370, 1.121) (*p* < 0.001 for interaction). Smoking assessment, statin prescribing, triple therapy prescribing, and blood pressure measurement significantly improved in intervention clinics relative to control, whereas cholesterol and glucose testing, and aspirin prescribing did not.

**Conclusions:**

It is feasible to use routine, paper-based, clinical records to evaluate essential CVD interventions in primary health care in Tajikistan. Adapted WHO PEN/HEARTS guidelines in the context of a complex intervention significantly improved blood pressure control after 12 months.

## Background

Non-communicable diseases (NCDs) are the leading cause of death worldwide and are particularly prevalent in Tajikistan. [1] The risk of premature mortality (death before the age of 70) from the four major NCDs (cardiovascular diseases, diabetes, cancer, and chronic respiratory disease) is 25 % with men being disproportionally affected. [2] This is in part due to the high population prevalence of tobacco and alcohol use among men, in addition to a 47 % prevalence of overweight in the adult population. [3] These trends also hold true in Tajikistan.

The detection and management of cardiovascular disease risk in primary health care is an urgent priority given the burden of cardiovascular disease (CVD) and the current suboptimal state of primary health care in Tajikistan. Population health data is scarce in Tajikistan but the best estimates to our knowledge, although not publicly available, are WHO STEPS survey data which estimates that 44.3 % of men and 24.7 % of women have never had their blood pressure measured and the prevalence of high CVD risk among adults aged 40–69 is 13.8 %. [3]

Early efforts in Tajikistan to build primary health care capacity and implement essential interventions for CVD in primary health care had mixed success.[4] From 2013 through to 2015 Tajikistan adapted and piloted the WHO Package of Essential NCD Interventions for Primary Healthcare in Low Resource Settings (WHO PEN). [5] WHO PEN provides an integrated approach, through the use of total cardiovascular risk assessment, to the detection and management of cardiovascular disease risk factors. While risk stratifying and targeting those at highest risk is a “best buy” intervention in terms of cost effectiveness, the initial piloting failed to achieve demonstrable health gains. [6] Some of the key shortcomings were that there was not support for primary health care workers following training, high turnover of staff, and no feedback on performance.

With renewed intent and led by the Ministry of Health and Social protection, in 2018 Tajikistan endeavoured to pilot and evaluate the newly developed HEARTS technical package, which aimed to provide a strategic approach to improving cardiovascular health including simplified clinical guidelines. [7] Together with the WHO Regional Office for Europe and the WHO Country Office of Tajikistan, the Ministry of Health and Social Protection convened a national steering group tasked with adapting, piloting, and evaluating the WHO PEN/HEARTS technical package with a particular focus on systems monitoring, building sustainable and scalable approaches, and integrating with other health systems reforms.

To achieve this, a complex intervention including a two-day training course on the prevention and management of CVD risk, clinical guidelines, paper-based decision support tools, and implementation guidance was developed and piloted in one region of Tajikistan. The aim of this study was to determine the feasibility of implementing and evaluating the complex intervention and its impact on the management of hypertension in primary health care. [3]

## Methods

The study protocol including methods has been previously published. [3] Herein we report the findings from the quantitative strand of a mixed methods study and the abbreviated methods below. Please refer to the published protocol for further methodological details and a broader overview of the study process. [3]

 The objectives were to: (1) determine the baseline performance of primary health care facilities; (2) to estimate the change in clinical practice over a 12 month period; and (3) to determine the feasibility of collecting quantitative data required for future studies of effectiveness. An additional objective, to assess the ability to train primary health care clinicians and implement Tajikistan-adapted WHO PEN/HEARTS tools, is not reported here and is addressed in the qualitative strand of the study design.

A pragmatic, sequential-mixed methods explanatory design was used. A single district (Shahrinav) was selected based on its nomination by the Ministry of Health, its year-round road access, accessibility from Dushanbe, and lack of exposure to previous interventions. Due to resource constraints and ongoing primary health care reform projects, it was not possible to work in more than one district.

All primary health care centres in Shahrinav were eligible, unless they met one or more exclusion criteria. The exclusion criteria were: [1] city health centre designation (these centres are not nationally representative of primary health care) [2] presence of narrow specialists (i.e. medical specialists other than family medicine) [3] clinics with a family doctor to patient ratio of 1:4000 or more or [4] clinics where information on the number of family doctors and/or family doctor to patient ratio is not available.[3] Included clinics were then randomized to either intervention or usual care arms using computer randomization.

While the full description has been previously published, [3] a brief description of the intervention components are as follows:

- One-page clinical protocols for hypertension and diabetes adapted from the WHO HEARTS technical package [7] and WHO PEN [8] in line with national guidelines.
- In-person two-day training integrating adult learning principles and focusing on practical clinical skills covering topics such as measurement and clinical management of CVD risk factors, task sharing, recorded keeping, and patient education material.
- Supportive supervision visits and peer review visits and discussion.
- Paper-based clinical decision support tools including WHO/ISH CVD risk charts, BMI charts, behavioural risk factor counselling.
- Quality improvement and management tools including quarterly monitoring visits using the HEARTS monitoring tool and direct support to improve paper based record keeping systems [7]..

The primary outcome was the proportion of hypertensive patients whose blood pressure was controlled, as defined by the number of patients with confirmed hypertension who visited the clinic at least once in the previous 12 months, whose most recent blood pressure reading was controlled (SBP < 140 and DBP < 90). A priori secondary outcomes have been previously published and a complete list of indicators are shown in Table 1. [3]

**Table 1:** Definitions of indicators in Tables 3 and 4

**Risk factors**	
Current smoker	Proportions of smokers. Patients were assumed to be non-smokers if smoking status was not specified.
Diabetes	Proportion of patients with a medical diagnosis^a^ of diabetes mellitus. Patients were assumed to be non-diabetic if diabetes status was not specified.
History of CVD	Proportion of patients with a medical diagnosis^b^ of cardiovascular disease
SBP/DBP	Mean based on the most recent systolic/diastolic blood pressure measurement^c^
**Process indicators**	
Smoking status recorded	Proportion of patients with smoking status recorded
BP measured regularly	Proportion of patients with two documented BP measurements^c^
Fasting glucose tested	Proportions of patients with fasting glucose tested
Total cholesterol tested	Proportions of patients with total cholesterol tested
WHO/ISH risk score documented	Proportions of patients aged 40 years or more with a WHO/ISH CVD risk score documented by health workers based on age, sex, BP, smoking status, diabtes status, and total blood cholesterol
WHO/ISH risk score calculatable	Proportions of patients aged 40 years or more with all risk factors (age, sex, BP, smoking status, diabetes status, and total blood cholesterol) recorded to calculate^d^ the WHO/ISH CVD risk score. Patients were assumed to be non-smoker if smoking status was not specified and to be non-diabetic if diabetes status was not specified. Predictions charts without total cholesterol were applied for those without measurement. The most recent BP and total cholesterol had to be during the last 24 months.
High-risk patients	Proportions of patients with a calculated^d^ WHO/ISH ≥ 30 % or/and a history of CVD
BP lowering drug prescribed	Proportions of patients with BP lowering drug prescribed
Statin prescribed for high-risk patients	Proportions of high-risk patients (calculated^d^ WHO/ISH ≥ 30 % or/and history of CVD
Aspirin prescribed for high-risk patients	Proportions of high-risk patients (calculated^d^ WHO/ISH ≥ 30 % or/and history of CVD) prescribed aspirin
Triple therapy prescribed for high-risk patients	Proportions of high-risk patients (calculated^d^ WHO/ISH ≥ 30 % or/and history of CVD) prescribed triple therapy (statin, aspirin, and blood pressure medication)
**Outcome indicator**	
BP at normal range	Proportion of patients whose most recent BP measurement^c^ was at normal range (SBP < 140 mmHg and DBP < 90 mmHg)
High-risk patients with BP at normal range	Proportion of high-risk patients (calculated^d^ WHO/ISH ≥ 30 % or/and history of CVD) whose most recent BP measurement^c^ was at normal range (SBP < 140 mmHg and DBP < 90 mmHg)
Low-risk patients with BP at normal range	Proportion of low-risk patients (calculated^d^ WHO/ISH < 30 % and no history of CVD) whose most recent BP measurement^c^ was at normal range (SBP < 140 mmHg and DBP < 90 mmHg)

*CVD* cardiovascular disease; *SBP* systolic blood pressure; *DBP* diastolic blood pressure; *BP* blood pressure; *WHO/ISH* risk score, World Health Organization/International Society of Hypertension cardiovascular risk score.

^a^ Diagnoses written either using ICD-10 codes or as names of diseases to the section of permanent diagnoses in the patient records are taken into account.

^b^ Diagnoses written either using ICD-10 codes or as names of diseases or events in the patient records are taken into account.

^c^ Measured during the last 12 months.

^d^ Calculated by the authors of this study.

 Two cross sectional samples were made, one before and one after implementation; patients from the first sample were not followed longitudinally. Each primary health care centre maintains a register of hypertensive patients and this was used for randomly sampling medical records. At baseline, the sample size was 50 records per clinic and this was adapted to include all patients on the register at follow-up because of low sample sizes at baseline. Inclusion criteria for records were that they were sampled from the hypertension register, that the patient had visited in the last 12 months, and that the patient was at least 18 years or older 12 months prior to the date of data extraction. Data were extracted into a standardized data collection form and entered into excel.

A 10 % point difference in the primary outcome between the intervention and control arms could be detected having 310 to 350 observations per arm at baseline and follow-up using a 0.05 type I error rate and 0.2 type II error rate. A target sample size of 400 per arm was therefore set to ensure ample data.

Age and sex adjusted logistic regression models were used for intervention and control clinics to determine changes between baseline and follow-up and to assess interactions between allocation group and time. Effect estimates were adjusted for age, sex, and clinic to account for clustering at the clinic level. For continuous variables, multivariate linear regression models were used. P-values of < 0.05 were considered statistically significant. WHO/ISH CVD risk scores were calculated using the whoishRisk package in R. [9]

The study team included local physicians, nurses, academics, WHO national office and European office representatives and worked in close collaboration with the Ministry of Health. A national team of experts adapted all intervention materials (e.g. clinical algorithms) and conducted in person training of health professionals with support from the entire study team. A small team of specially trained national experts used standardized data extraction sheets to record pre-specified routine data that were used to calculate both process and outcome indicators. [3]

## Results

In Shahrinav district, 19 clinics met inclusion criteria and ten were randomized to intervention and nine were randomized to usual care. In total, 15 doctors and 105 nurses were trained. At baseline, 260 and 207 unique patient records were included in the intervention and control group sample, respectively (Table 2). At follow-up, this increased to 374 and 244 unique records, respectively. This translates to a total sample of 1,085 with 467 at baseline and 618 at follow-up. Age distributions were similar between intervention and control at baseline and follow-up. The proportion of newly diagnosed hypertension patients increased significantly in the intervention clinics (from 25 to 47 % *p* < 0.001) but not the control clinics (from 14 to 20 % *p* = 0.091); however this increase was not significantly different in the intervention compared to the control (*p* = 0.184 for interaction).

**Table 2:** Demographic characteristics of patients at baseline and 1-year follow-up data collection

**Characteristics**	**Intervention**		**Control**	
	**Baseline (*N* = 260)**	**Follow-up (*N* = 374)**	**Baseline (*N* = 207)**	**Follow-up (*N* = 244)**
Sex				
Female, % (n)	53.1 (138)	63.9 (239)	55.6 (115)	62.3 (152)
Male, % (n)	46.9 (122)	36.1 (135)	44.4 (92)	37.7 (92)
Age, median (IQR)	62 (57–69)	62 (57–68)	64 (56–71)	63.5 (58–70)
29–49 years, % (n)	6.2 (16)	8.0 (30)	5.3 (11)	4.1 (10)
50–59 years, % (n)	30.8 (80)	30.2 (113)	32.9 (68)	30.7 (75)
60–69 years, % (n)	40.0 (104)	40.9 (153)	32.9 (68)	37.3 (91)
70–79 years, % (n)	15.0 (39)	14.2 (53)	21.7 (45)	20.9 (51)
80 + years, % (n)	8.1 (21)	6.7 (25)	7.2 (15)	7.0 (17)

*IQR* interquartile range

There was no significant difference between intervention and control groups with respect the change in proportion of those who smoke, had diabetes, or a history of CVD (Table 3). There was a statistically significant reduction in systolic and diastolic blood pressure among the intervention group but not the control group (*p* < 0.001). In the intervention group, mean systolic blood pressure was reduced by 8.51 mmHg (95 % CI -10.65, -6.43) (Table 3). With respect to CVD risk factors among those sampled, too few patients had documented total cholesterol or fasting blood glucose to calculate a reasonable estimate.

**Table 3:** Risk factors for intervention and control clinics at baseline and 1-year follow-up

**Risk factors**		**Baseline (***N*** = 467)**		**Follow-up (*N* = 618)**		**Baseline vs. Follow-up Difference**^**a**^		**Interaction**^**b**^
						**OR (95 % CI)/difference in means**	***P*-value**	***P*-value**
**Intervention**								
Current smoker, % (n/N)		5.4 (14/260)		7.2 (27/374)		2.059 (0.933–4.545)	0.074	0.829
Diabetes, % (n/N)		15.8 (41/260)		19.3 (72/374)		1.354 (0.872–2.102)	0.177	0.973
Newly diagnosed hypertension, % (n/N)		25.4 (66/260)		47.1 (176/374)		2.520 (1.743–3.643)	< 0.001	0.184
History of CVD, % (n/N)		20.0 (52/260)		13.1 (49/374)		0.653 (0.397–1.074)	0.093	0.537
SBP, mean ± SD		146.52 ± 14.25		137.80 ± 12.07		-8.51 (-10.65–6.37)	< 0.001	< 0.001
DBP, mean ± SD		91.80 ± 9.04		86.61 ± 7.85		-5.30 (-6.65–3.96)	< 0.001	< 0.001
**Control**								
Current smoker, % (n/N)		6.8 (14/207)		9.0 (22/244)		1.869 (0.759–4.598)	0.173	
Diabetes, % (n/N)		18.8 (39/207)		23.4 (57/244)		1.371 (0.843–2.231)	0.204	
Newly diagnosed hypertension, % (n/N)		14.0 (29/207)		19.7 (48/244)		1.565 (0.931–2.631)	0.091	
History of CVD, % (n/N)		41.5 (86/207)		37.7 (92/244)		0.767 (0.509–1.156)	0.205	
SBP, mean ± SD		142.28 ± 16.58		141.49 ± 14.21		-0.52 (-3.36-2.32)	0.721	
DBP, mean ± SD		89.83 ± 8.89		89.85 ± 9.29		0.07 (-1.63-1.77)	0.932	

*CVD* cardiovascular disease; *SBP* systolic blood pressure; *DBP* diastolic blood pressure; *SD* standard deviation; *OR* odds ratio; *CI* confidence interval

^a^ Adjusted for age, gender, and clinic.

^b^ Interaction for intervention and control clinics adjusted for age, gender, and clinic.

**Fig. 1:**
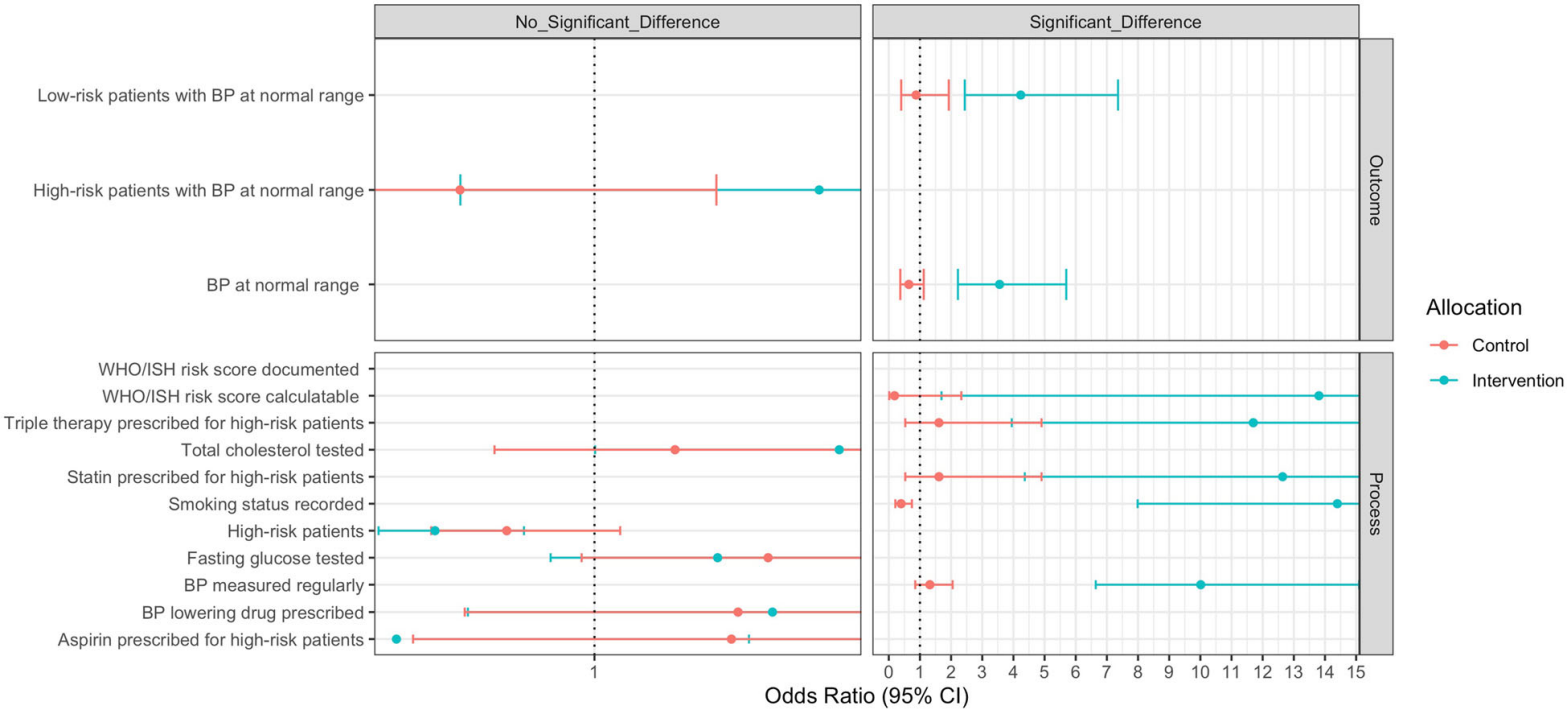
Odds of achieving outcomes (odds ratio with 95 % CI) at 1-year follow-up compared to baseline for both intervention and control clinics

Among the intervention group, the proportion of hypertensive patients with blood pressure in normal range (primary outcome) significantly improved from baseline to follow-up (OR 3.556, 95 % CI 2.219, 5.696); there was no significant change in the control group (OR 0.644, 95 % CI 0.370, 1.121) (Table 4; Fig. 1). The improvement in the intervention groups was statistically significantly larger than the control (p for interaction < 0.001) (Table 4). This was also true for blood pressure control among patients with low WHO/ISH CVD risk scores but not for those with high CVD risk scores (Table 4; Fig. 1).

**Table 4:** Process and outcome indicators at baseline and 1-year follow-up

**Process and outcome indicators**	**Baseline**	**Follow-up**	**Baseline vs. Follow-up Difference**^**a**^		**Interaction**^**b**^
	**% (n/N)**	**% (n/N)**	**OR (95 % CI)**	***P*-value**	***P*-value**
**Intervention**					
** Process indicators**					
Smoking status recorded	61.5 (160/260)	90.4 (338/374)	**14.397 (7.987–25.886)**	< 0.001	< 0.001
BP measured regularly	25.0 (65/260)	71.4 (267/374)	10.013 (6.642–15.094)	< 0.001	< 0.001
Fasting glucose tested	13.1 (34/260)	16.3 (61/374)	1.393 (0.860–2.255)	0.178	0.800
Total cholesterol tested	8.1 (21/260)	12.3 (46/374)	1.781 (1.002–3.165)	0.049	0.423
WHO/ISH risk score documented	0.0 (0/259)	79.7 (295/370)	-	-	-
WHO/ISH risk score calculatable	96.1 (249/259)	99.7 (369/370)	13.805 (1.692-112.649)	0.014	0.004
High-risk patients	26.5 (69/260)	15.0 (56/374)	0.491 (0.311–0.775)	0.002	0.216
BP lowering drug prescribed	96.5 (251/260)	97.1 (363/374)	1.568 (0.596–4.124)	0.362	0.910
Statin prescribed for high-risk patients	23.2 (16/69)	78.6 (44/56)	12.640 (4.365–36.599)	< 0.001	0.001
Aspirin prescribed for high-risk patients	85.5 (59/69)	87.5 (49/56)	**0.368 (0.091–1.493)**	**0.162**	0.213
Triple therapy prescribed for high-risk patients	23.2 (16/69)	76.8 (43/56)	11.699 (3.947–34.679)	< 0.001	0.001
** Outcome indicators**					
BP at normal range	14.8 (34/229)	32.5 (118/363)	3.556 (2.219–5.696)	< 0.001	< 0.001
High-risk patients with BP at normal range	15.8 (9/57)	20.4 (11/54)	1.717 (0.572–5.158)	0.335	0.133
Low-risk patients with BP at normal range	14.5 (25/172)	34.6 (107/309)	4.235 (2.438–7.358)	< 0.001	0.002
**Control**					
** Process indicators**					
Smoking status recorded	73.4 (152/207)	68.0 (166/244)	**0.395 (0.210–0.743)**	0.004	
BP measured regularly	62.3 (129/207)	68.9 (168/244)	1.319 (0.849–2.049)	0.218	
Fasting glucose tested	22.7 (47/207)	30.7 (75/244)	1.554 (0.959–2.519)	**0.074**	
Total cholesterol tested	12.1 (25/207)	15.2 (37/244)	1.257 (0.681–2.321)	0.464	
WHO/ISH risk score documented	0.0 (0/203)	0.4 (1/242)	-	-	
WHO/ISH risk score calculatable	99.5 (202/203)	98.3 (238/242)	0.185 (0.015–2.328)	0.192	
High-risk patients	46.9 (97/207)	41.8 (102/244)	0.720 (0.479–1.082)	0.114	
BP lowering drug prescribed	94.7 (196/207)	95.9 (234/244)	1.458 (0.586–3.628)	0.418	
Statin prescribed for high-risk patients	13.4 (13/97)	15.7 (16/102)	1.610 (0.529–4.903)	0.402	
Aspirin prescribed for high-risk patients	89.7 (87/97)	92.2 (94/102)	1.437 (0.421–4.903)	0.562	
Triple therapy prescribed for high-risk patients	13.4 (13/97)	15.7 (16/102)	1.610 (0.529–4.903)	0.402	
** Outcome indicators**					
BP at normal range	17.8 (36/202)	13.2 (31/234)	0.644 (0.370–1.121)	0.120	
High-risk patients with BP at normal range	22.1 (21/95)	13.0 (13/100)	0.571 (0.235–1.389)	0.216	
Low-risk patients with BP at normal range	14.2 (15/107)	13.4 (18/134)	0.877 (0.399–1.926)	0.744	

*BP* blood pressure, *WHO/ISH* risk score World Health Organization/International Society of Hypertension cardiovascular risk score; *OR* odds ratio; *CI* confidence interval.

^a^ Adjusted for age, gender, and clinic

^b^ Interaction for intervention and control clinics adjusted for age, gender, and clinic

Of the process indicators, five significantly improved among the intervention group compared with control group. These were: the ability to calculate WHO/ISH CVD risk score, triple therapy prescribing for high risk patients, statin prescribing for high risk patients, recording of smoking status, and regular measurement of blood pressure (Table 4; Fig. 1). The remaining process indicators (total cholesterol testing, fasting glucose testing, prescription of blood pressure lowering medication, aspirin prescribing for high-risk patients), were not significantly different between intervention and control groups. At baseline, none of the records contained a documented WHO/ISH risk score. This increased to 79.7 % (295 of 370 records) at follow-up in the intervention group, in contrast to only 0.4 % (1 of 242 records) in the control group. The proportion of patients with high CVD risk decreased significantly in intervention clinics, but was not significantly different than the change seen in control clinics (Table 4; Fig. 1).

## Discussion

Baseline and follow-up collection and analysis of data from primary health care centres in Tajikistan demonstrated that it is feasible to collect quantitative data required for studies of effectiveness from routine clinical data. Furthermore, we were able to determine the baseline performance of primary health care services with respect to essential interventions for the management of hypertension and prevention of CVD, and their change over time. The intervention significantly improved blood pressure control, assessment of cardiovascular risk factors including blood pressure measurement and smoking status, and prescription of statins and triple therapy. It also significantly increased the proportion of newly diagnosed hypertension patients. It did not improve the testing rates of total cholesterol, fasting glucose tests, prescription of aspirin or blood pressure lowering drugs.

Measurement of biochemical risk factors is a basic part in the standard of care in the detection and management of NCDs yet the measurement of total cholesterol and fasting glucose were profoundly suboptimal. [10, 11] This is likely because of low availability and accessibility of these tests in primary health care, including having to pay out-of-pocket. Despite this, in resource limited settings it is still possible to CVD risk stratify individuals without access to laboratory testing and this was seen in the intervention group but not the control group.[9, 12, 13] Documentation of WHO/ISH CVD risk score improved from 0 to 79.7 % in the intervention group illustrating a drastic uptake of CVD risk assessment independent of an increase in cholesterol testing. This is possible since there are cholesterol-independent WHO/ISH CVD risk charts.

Furthermore, using the cholesterol-independent WHO/ISH risk charts, we were able to calculate WHO/ISH risk scores for nearly 100 % of patients in both intervention and control groups. This demonstrated an important finding – while biochemical risk factor assessment is underutilized, it is still possible to translate existing clinical data into meaningful total cardiovascular risk scores without additional need for testing using WHO/ISH CVD risk charts. This is was true for both intervention and control groups, implying that given the appropriate training and access to CVD risk charts, clinicians can quickly implement CVD risk scoring into their existing clinical workflow.

There was no significant difference in the rate of blood pressure lowering medication prescription between intervention and control groups, which was explained by the nearly perfect performance of this indicator at baseline. In the intervention and control groups, 96.5 and 94.7 % of hypertensive patients, respectively, were prescribed blood pressure lowering medication. The observed effect on blood pressure reduction in intervention clinics may therefore be more strongly associated with the behavioural risk factor counselling and patient education materials that were part of the intervention.[14] The prescription of statins, however, significantly improved in the intervention group but not in the control group without changes in the rate of cholesterol testing. This is likely due to the fact that in the absence of cholesterol testing, statins can according to WHO PEN protocol be prescribed to those with high CVD risk scores including cases of secondary prevention.[5] Of course, it is unclear the degree to which these prescriptions are filled and the proportion who achieve medication adherence.

Results show a significant decline in blood pressure levels and proportion of patients with elevated blood pressure in intervention clinics compared with control clinics. However, no significant improvement was observed in prescription rates of blood pressure lowering medication, which was very high already at baseline. There may have been changes to the dosing of medication according to the HEARTS protocols, but unfortunately drug dosage information was not collected. Increased medication adherence due to improved patient counselling may have also played a role. The decline in observed mean blood pressure in the intervention group can also be partly explained by the increased number of newly diagnosed patients with overall lower (but raised) blood pressure. The proportion of newly diagnosed hypertension patients increased significantly in the intervention clinics, which may be accounted for by increased screening. Forthcoming qualitative research will seek to explain these findings.

Previous research examining the quality of hypertension care in the Khatlon and Sogd oblasts of Tajikistan found persistent gaps in the detection, diagnosis, and outcomes of patients with hypertension.[15] In particular, it was estimated that only 5 to 10 % of hypertensive patients actually receive a diagnosis. This is consistent with our findings in that the number of registered hypertensive patients in the Shahrinav oblast was far below the expected, which contributed to our lower than expected sample size. This is in part explained by insufficient supply of equipment (e.g. sphygmomanometers), high case load of primary health care workers, and lack of awareness among health administrators.[15] However, according to this evaluation study the detection rate of hypertension seems to have improved in intervention clinics in Shahrinav as both the number of patients recorded having hypertension as well as newly diagnosed patients increased.

The aspects of our intervention that may have contributed to the improvement in blood pressure control and several surrogate markers along the pathway of care, include a focus on known challenges to implementation and building on the previous work done nationally with WHO PEN. These lessons and experiences were used to adapt the HEARTS technical package to best meet known challenges to successful implementation. These include: using evidence-based, simplified, protocols designed for primary health care, conducting integrated training of doctors and nurses with task sharing and quality of care as part of the curriculum, the provision of decision support tools, an emphasis on grass roots quality improvement, and monitoring and feedback for quality improvement. [16] Without a comprehensive approach, however, taking into account organization processes, capacity to implement simplified clinical guidelines, the provision of essential equipment, medicines, and diagnostic tests further improvements may be limited. [17] Focus should be the continued prioritization of NCDs for public health intervention, optimizing clinical guidance for primary health care, engagement of key stakeholders, generating a local (i.e. Tajikistan) evidence base, and ensuring quality improvement while mainstreaming. [16]

Improvements in blood pressure control were also observed in the Republic of Moldova after the implementation of adapted WHO PEN guidelines and structured training for health workers. Compared to baseline, significant improvements were seen in intervention clinics at one year follow-up for patients with blood pressure at normal range, hypertensive patients with blood pressure at normal range, and patients with elevated blood pressure. Similar to our study, no significant change in aspirin prescriptions for CVD patients were observed. In contrast to our study, the prescriptions of statins for CVD patients did not change in intervention clinics and even deteriorated in control clinics.[18].

It was noted that the proportion of women increased in the follow-up (random) sample in both intervention and control clinics. We speculate that home visit programs that were not a part of our study that may have biased the clinic registers toward women over time as men are often not in the home during the day or are away for extended periods of time for work. This, and other important questions such as the adoption and perception of the intervention across the ten sites, the use of standard clinical algorithms and guidance, and perceived barriers will be examined in the qualitative arm of this study and is beyond the scope of this manuscript.

### Strength and Limitations

This study is strengthened by its a *priori* publication of methods and its ability to be reproduced and scaled within the constraints of the existing Tajik health system. This work will be further strengthened by the planned qualitative research as part of a broader sequential explanatory mixed-methods design. There were four main limitations. First, the targeted sample size was not fully achieved as there were not enough registered hypertension patients to meet our target sample size. The study is therefore underpowered for some outcomes. Outcomes which did not show a statistically significant difference between intervention and control groups should therefore be judged in light of the possibility that if a difference did exist, this study may not detect it. Second, due to limited health record infrastructure, patients had to be sampled from the hypertension register so we were unable to assess patients without a formal diagnosis of hypertension. Third, for pragmatic reasons including the rugged and vast geography of Tajikistan, only one health region was included in the study and this may limit the generalizability of the findings although it is not known to be an outlier in terms of health system development or performance. Fourth, data collectors were unable to be blinded to allocation during the follow-up data collection due to the nature of the intervention.

## Conclusions

It is feasible to use routine, paper-based, clinical records to evaluate essential CVD interventions in primary health care in Tajikistan. Pilot implementation of adapted WHO and HEARTS guidelines, including the systems for monitoring, demonstrated a significant impact on blood pressure control among hypertensive patients after 12 months, as well as several process indicators including CVD risk assessment. Continued monitoring and evaluation will be required as this intervention is scaled nationally.

## Data Availability

The datasets used and/or analysed during the current study are available from the corresponding author on reasonable request.

## Abbreviations

BMI: Body mass index
CI: Confidence interval
CVD: Cardiovascular disease
ISH: International society of hypertension
NCD: Non-communicable disease
OR: Odds ratio
PEN: Package of essential NCD interventions for low-resource settings
WHO: World health organization
